# Vitamin D status and determinants in Indian children and adolescents: a multicentre study

**DOI:** 10.1038/s41598-022-21279-0

**Published:** 2022-10-06

**Authors:** Anuradha Khadilkar, Neha Kajale, Chirantap Oza, Rashmi Oke, Ketan Gondhalekar, Vivek Patwardhan, Vaman Khadilkar, Zulf Mughal, Raja Padidela

**Affiliations:** 1 Department of Growth and Endocrinology, Hirabai Cowasji Jehangir Medical Research Institute, Jehangir Hospital, 32 Sassoon Road, Pune, 411 001 India; 2 Interdisciplinary School of Health Sciences, Savitribai Phule University, Pune, India; 3 Department of Paediatric Endocrinology, Royal Manchester Children’s Hospital, Manchester University, NHS Foundation Trust, Manchester, UK

**Keywords:** Biological techniques, Endocrinology, Health care

## Abstract

Studies performed on Indian children to assess vitamin-D status have been on small sample sizes, limited to specific geographical locations and used non-standard methods to measure 25(OH)D_3_. This multicentre study assessed 25(OH)D_3_ concentrations from dried blood spots (DBS) in 5–18-year-old Indian children and adolescents using a standardized protocol and identified factors contributing towards vitamin D deficiency. Cross-sectional, observational school-based study was conducted by multi-stage stratified random sampling. A city and nearby village were selected from 6 Indian states covering wide geographical areas. Demography, anthropometry, body-composition, dietary-intakes and DBS samples were collected. 25(OH)D_3_ was assessed from DBS using Liquid chromatography with tandem-mass spectrometry. Vitamin-D status was assessed in 2500 children; with additional data collected on a subset (n = 669) to assess predictors. Mean vitamin-D concentration was 45.8 ± 23.9 nmol/L, 36.8% of subjects had sufficient vitamin-D (> 50 nmol/L); rural subjects and boys had higher concentrations (*p* < 0.05). On regression analysis, younger age, female-gender, overweight and urban residence significantly contributed to deficiency. More than half the Indian children/adolescents were vitamin-D deficient or insufficient. Our study reinforces vitamin-D deficiency as a major public health problem and the need for supplementation, food fortification and educating the population as initiatives required to improve sufficiency status.

## Introduction

Vitamin D is a secosterol with a major role in maintaining calcium and phosphorus homeostasis. Vitamin D affects calcium homeostasis by its action on the kidney, intestine and bone^1^. Vitamin D also has direct and indirect effects on growth plates, bone and bone cells^1^. The extra-skeletal role of vitamin D has been suggested in various organs and ailments like skin (psoriasis, skin cancer), muscle function, cardiomyopathy, immune system (respiratory tract infection, inflammatory bowel disorder, allergy), colorectal carcinoma, cardiovascular risk factors (hypertension, diabetes, obesity, metabolic syndrome), neurological disorders and reproductive function^1^. It is generally accepted that serum 25-hydroxyvitamin D (25(OH)D) is a reliable measure of an individual’s vitamin D status. Serum total 25(OH)D concentration is the sum of the 25(OH)D_3_ and 25(OH)D_2_ concentrations. Various methods such as Radioimmunoassay (RIA), Chemiluminescence immunoassay, Enzyme-Linked Immunosorbent Assay (ELISA), and protein binding assays are used for measurement of 25(OH)D concentrations. However, High-Performance Liquid Chromatography (HPLC) or tandem mass spectrometry are considered to be the gold standard for the assessment of 25(OH)D_3_^2^. Thus, liquid chromatography-tandem mass spectrometry (LC–MS/MS) is the widely accepted reference method for 25(OH)D measurement, however, it requires expensive equipment and expertise and is seldom used in low- or middle-income countries such as India^3^.

The Indian Academy of Paediatrics Guidelines and the Global Consensus on Prevention as well as Management of Nutritional Rickets recommend that 25(OH)D concentrations of over 50nmols/L (20ngm/ml) are sufficient, between 30 and 50 nmols/L (12–20 ngm/ml) are insufficient and below 30 nmols/L (12 ngm/ml) are in the deficiency range in children and adolescents^3,4^. A systematic review published in 2014 suggests that vitamin D deficiency and insufficiency are a major public health problem globally irrespective of age, even in populations residing in countries where it is assumed that UV radiation is adequate and in industrialized countries where fortification has been implemented for years^5^. It is estimated that about 1 billion people across all ethnicities and age groups have low vitamin D concentrations worldwide^6^. In India, approximately 490 million people are vitamin D deficient of which 31% are children and adolescents^7^. India is a large country and in most published reports, participants are confined to one district or specific populations. Further, studies have been performed on relatively small sample sizes with differences in methods of vitamin D concentration estimation such as radioimmunoassay, ELISA, chemiluminescence etc. The prevalence of vitamin D deficiency has been also been described using different cut-offs, thus making interpretation of results difficult.

High prevalence of vitamin D deficiency in a sun-rich country like India (Latitude—8° 4′–37° 6′ N most of India has adequate Ultraviolet B radiation (UVB) throughout the year) has been reported due to several reasons including inadequate exposure to sunlight, dietary factors (inadequate vitamin D and calcium intake, high phytates and phosphates, intake of caffeine, high prevalence of lactose intolerance), skin pigmentation, pollution hampering penetration of Ultraviolet rays, genetic polymorphisms and body fat percentage^8,9^. However, the contribution of various determinants that influence vitamin D concentrations in the Indian paediatric population is underreported. Taken together, given the importance of vitamin D status in children and adolescents and the reported prevalence of vitamin D deficiency in India, we undertook this prospective cross-sectional study to assess vitamin D status and identify determinants of vitamin D deficiency (VDD) in Indian children and adolescents. We conducted this multicentre study in 6 states covering a wide geographical area of India. To determine vitamin D status, a previously standardised^10^, LC–MS/MS based method for estimating 25-hydroxy-vitamin D3 (since in Indians 25(OH)D_3_ contributes to > 90% of total 25(OH)D concentrations) measurements from dried blood spots was used^8,10^. Our specific objectives were to assess vitamin D concentrations (25(OH)D_3_) from dried blood spots in 5–18-year-old Indian children and adolescents from six states of India and to identify factors (demographic, anthropometric and lifestyle) affecting vitamin D deficiency and insufficiency in Indian children and adolescents.

## Methods

### Study design and subjects

This was a multicentre, cross-sectional, observational school-based study; data collection was performed from July 2016 to October 2017. Sampling was carried out by adopting a multi-stage stratified random sampling procedure as described earlier^11,12^. Briefly, of the Indian states, 6 states namely Maharashtra, Gujarat, Chhattisgarh, Assam, Tamil Nadu and Punjab were randomly selected and from each state, a city and a nearby village were randomly selected. The selected cities were Pune (18.5° N), Bilimora (20.7° N), Raipur (21.2° N), Diphu (25.8° N), Chennai (13.0° N) and Mohali (30.7° N) while the selected nearby villages were Ranjangaon, Gandevi, Kurud, Manja, Urapakkam and Lalru from Maharashtra, Gujarat, Chhattisgarh, Assam, Tamil Nadu and Punjab, respectively. Lastly, a list of schools from selected centres was made and of hundred schools approached, the forty schools that gave permission were included in the study (schools mainly disagreed because they could not invest the time required for the study). All methods were carried out in accordance with declaration of Helsinki for biomedical research involving human subjects. Ethics committee approval was obtained from the Institutional ethics committee namely “Ethics committee Jehangir Clinical Development Center Private Limited, Pune, Maharashtra (EC registration number—ECR/352/Inst/MH/2013)” (Approval dated 21st June 2016). Health authorities, schools and parents/legal guardian gave written informed consent for all participating subjects and assent was obtained from all children (older than 7 years). All children were examined by a paediatrician and children's medical records were reviewed and those suffering from chronic disorders or disorders likely to affect calcium and vitamin D metabolism including malabsorption and those receiving calcium or vitamin D supplements were excluded.

### Sample size

As per previously reported studies^13^, considering average prevalence of vitamin D deficiency (VDD) to be about 60%, using the formula [z^2^*P(1 − P)/d^2^), where z = 1.96, and d = 5%, sample size per region was calculated as “n = 370 children/region”. Thus, at least 2200 subjects were required to assess VDD in 6 regions of India. Post hoc power analysis using G power (software version 3.1.9.2) indicated that sample of a subset (n = 669) was sufficient to achieve the power of the study (1 − beta error probability) = 0.83 at 0.05 level of alpha, with two-tailed logistic regression model (Z test family), and age/other factors [OR = 0.754 (0.671–0.841)] as significant predictors of VDD.

### Demographic data

Information about the date of birth, socio-demographic data, sunlight exposure and usage of sunscreens and past medical history was collected using a pre-validated questionnaire and cross-verified from school records^14^.

### Anthropometry

Height (Seca Portable stadiometer, Hamburg, Germany up to 0.1 cm accuracy) and body weight (Seca 876 Flat scale, Hamburg, Germany, up to 100 g accuracy) were measured using standard protocols. Body mass index (BMI) was computed using the following formula: BMI = weight (kg)/height (m)^2^. Triceps skinfold thickness (TSFT) was measured using a Harpenden calliper to the nearest mm on the non-dominant hand using standard protocols. Subsequently, the height, weight, TSFT and BMI were converted to Z scores using Indian reference data^14,15^.

### Body composition

Fat mass, fat-free mass and total body water were assessed using Bioelectrical Impedance Analyzer (BIA), (Tanita Model BC420MA) after a minimum of 3 h of fasting and voiding before measurements in a standing position^16^. Z scores for fat percentage and muscle mass percentage were calculated using Indian reference data^17^.

### Dietary intakes

Dietary intakes were recorded by trained nutritionists using the 24-h dietary recall method administered over 2 non-consecutive days, including a holiday or Sunday. For estimation of food intake, participants were interviewed in Hindi, English or the local language using standard cups and spoons and the multiple pass method. Nutrient intakes were then estimated using the C-Diet software based on the nutritive values of raw and cooked foods^18,19^. Calcium density was computed based on calcium intake in mg/100 cal consumed.

### Dried blood spot collection and vitamin D estimation

Blood collection was performed with the help of trained phlebotomists on Whatman filter paper no. 903 without any pre-treatment. Each child was made to sit comfortably and explained the protocol before the procedure. A spirit swab (70% ethyl alcohol) was used to clean the chosen finger and then pricked with a sterile single-use safety lancet. After discarding the first drop, further drops were spotted on a pre-labelled filter paper. After spotting 3–4 drops, the filter papers were air-dried and then packed separately in plastic bags with a 1gm desiccant bag. All the individually packed bags were then kept in black sealed labelled plastic bags to avoid exposure to sunlight. Each bag was placed in a − 80 °C freezer till further analysis. The DBS samples were extracted by the liquid–liquid extraction method using hexane for separation. The extracted samples were separated using ultra-high-performance liquid chromatography (UHPLC) and vitamin D (25(OH)D_3_) content was assessed using the LC–MS/MS system Shimadzu 8045 triple quadrupole mass spectrometer (MS) [Shimadzu Corporation, 2019, Japan] with the electrospray ionization (ESI) source in positive ion polarity mode fitted with Nexera X2 LC-30A UHPLC system as previously described^10^. All the samples were tested on the same machine in the same lab by the same trained lab personnel. The intra and inter-assay coefficient of variation was 7% and 8% respectively^20^.

### Data entry and warehousing

All data were double data entered in MySQL and errors were trapped using range checks. The dataset was then examined for consistency using measures of central tendency, ranges as well as distributions of continuous variables. Frequency tables were created for categorical variables to identify outliers and data inconsistencies.

### Statistical analysis

SPSS software for Windows (version 26.0, IBM statistics data editor, IBM Corp., released 2018. IBM SPSS Statistics for Windows, Version 26.0. Armonk, NY: IBM Corp) was used for data analysis; StataIC12 was used for tests of proportion. Variables were checked for normality; normally distributed variables were expressed as mean ± SD, and non-normally distributed variables were reported as median (IQR). Appropriate tests such as independent sample t-tests were used to assess the differences among urban and rural children for anthropometry, 25(OH)D_3_ concentrations, nutrient intakes, and body composition parameters for normally distributed data reported as mean ± SD. Mann Whitney-U test was used to analyse the differences in parameters that were non-normally distributed reported as median (IQR). ANOVA was used to assess the interstate differences separately for boys and girls. We also report survey-weighted parameters stratified by states and urban/rural settings using state-wise census 2011 data. From Census 2011, respective age, gender, and region-wise population data were used for obtaining a proportion factor corresponding to sample data for survey weighting procedure using the SPSS software^21^. A multinomial logistic regression analysis model was used for assessing the factors affecting 25(OH)D_3_ status. Coded Vitamin D deficiency and vitamin D insufficiency were used as dependent variables with reference to vitamin D sufficiency in the model^3^. Modifiable factors [such as sunlight exposure, dietary calcium intake, body composition parameters (BMI and TSFT)] and nonmodifiable factors e.g., age, gender, area of residence and socioeconomic status were used as independent predictors in the model. Variation across states was calculated by the two-sample proportion calculator using StataIC12. The level of significance was set at *p* < 0.05.

## Results

**Figure 1:**
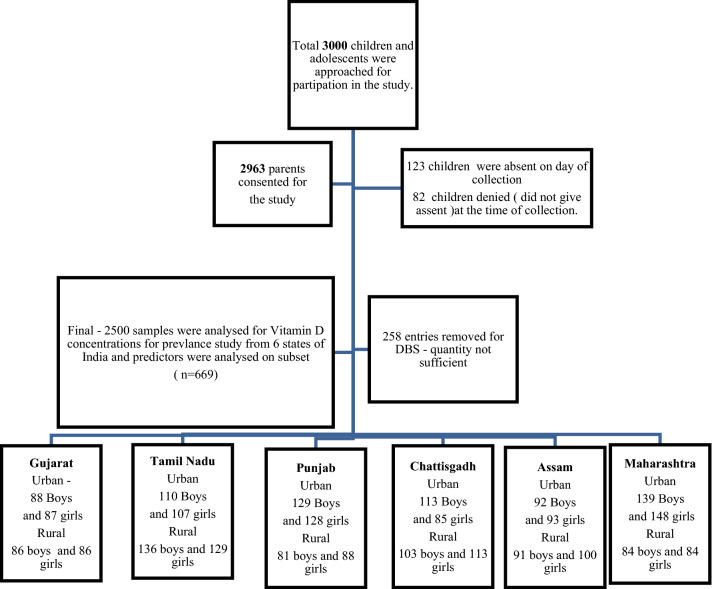
Flow diagram of study participants.

The study included a total of 2500 children, out of which 1252 (50.0%) were boys, 1248 (50.0%) girls and 1319 boys and girls (52.7%) belonged to urban areas; none of the study participants had a history of usage of sunscreens; latitudes of all the states ranged from a minimum of 13°–30.7° N. The total number of participants from each state were as follows: Gujarat—347 (13.9%), Tamil Nadu—482 (19.3%), Punjab—426 (17.1%), Chhattisgarh—414 (16.6%), Assam 376 (15.1%) and Maharashtra—455 (18.1%). The age and area wise number of participants from each state are illustrated in Fig. 1.

**Figure 2:**
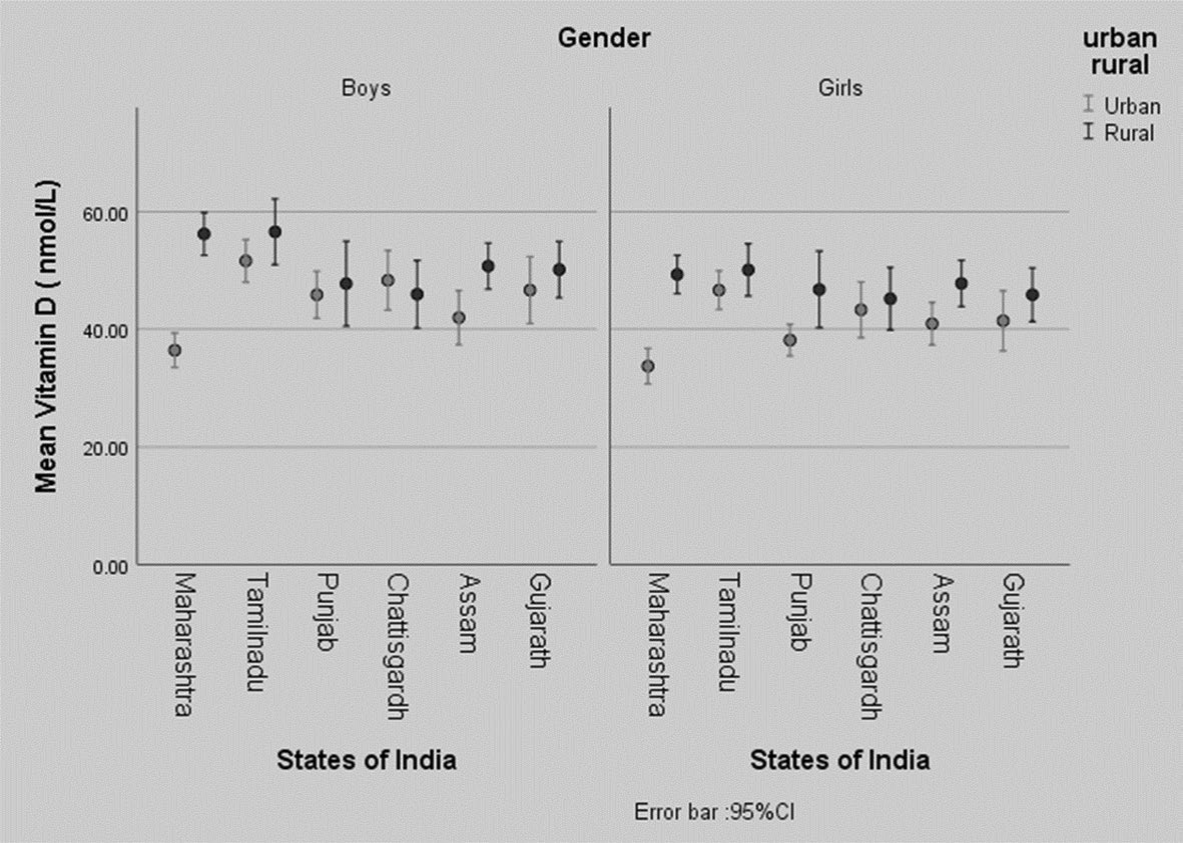
State wise comparison of mean (± SE) Vitamin D concentrations stratified by urban rural residence and gender. *p* values are displayed in Table 1 between urban and rural counterparts.

Table 1 describes the gender and geography wise mean anthropometric parameters and vitamin D concentrations of subjects enrolled in the study. The mean (± SD) age and gender standardized Z-scores for height, weight and BMI of the study population were 11.5 ± 3.1 years, − 0.4 ± 1.0, − 0.5 ± 1.0 and − 0.5 ± 1.0 respectively. Urban subjects were taller and heavier than their rural counterparts except for subjects from Tamil Nadu (where there were no differences in the urban and rural subjects, *p* > 0.05) and girls from Punjab (no differences in urban and rural girls, *p* > 0.05). The overall mean vitamin D concentration of the study population was 45.8 ± 23.9 nmol/l. Gender-wise, state-wise and area-wise vitamin D deficiency and insufficiency are illustrated in Fig. 2 (mean 25(OH)D_3_ concentrations). Overall, mean vitamin D concentrations of both urban and rural boys were significantly higher than that of their female counterparts (*p* < 0.05). Vitamin D concentrations of the rural population were significantly (*p* < 0.05) higher than their urban counterparts except for in Chhattisgarh where the urban boys had higher mean vitamin D concentrations than their rural counterparts but the difference was insignificant (*p* > 0.05). The urban Maharashtrian boys and girls had significantly lower mean vitamin D concentrations than their corresponding counterparts except for Assamese boys (urban boys from Maharashtra and Assam did not have different vitamin D concentrations, *p* > 0.05) and Punjabi girls (there were no significant differences in vitamin D concentrations between Punjabi and Maharashtrian girls, *p* > 0.05). No significant differences (*p* > 0.05) were observed in mean vitamin D concentrations of rural boys and girls with their respective counterparts except for significantly higher vitamin D concentrations of rural Tamil boys than that of rural boys of Chhattisgarh.

**Table 1:** State, region (urban/rural) and gender-wise demographic and anthropometric characteristics with vitamin D concentrations of the study population.

States	Gujarat		Tamil Nadu		Punjab		Chhattisgarh		Assam		Maharashtra	
Latitude	19.9–24.8° N		7.9–13.6° N		29.3–32.3° N		18.2–23.4° N		24.8–28.2° N		15.3–22.6 ^0^ N	
	Urban	Rural	Urban	Rural	Urban	Rural	Urban	Rural	Urban	Rural	Urban	Rural
**Boys**												
Age (years)	**10**.6 ± 3*^t,p,c^	11.6 ± 2.9	11.9 ± 2.9 g	11.4 ± 3.5	12.6 ± 3.6*^m,a,g^	11.6 ± 3.2	12.1 ± 2.4*^g,^	12.1 ± 3.6 m	11 ± 3 ^a^	11.1 ± 3.4	11.2 ± 2.7^p^	10.5 ± 2.7^c^
Height Z-Score	− 0.4 ± 1.1*^c,m^	− 0.7 ± 1.1^t,c^	− 0.1 ± 1	0.1 ± 1.3^m,p,c,a,g^	− 0.4 ± 1.1*^m,c^	− 0.8 ± 1^t^	0.1 ± 1* ^**p,a,g**^	− 1.3 ± 1 ^t,a,g^	− 0.5 ± 1*^m,c^	− 0.8 ± 1.1^t,c^	0.1 ± 1*^p,a,g^	− 1.1 ± 0.9^t^
Weight Z-Score	− 0.4 ± 1.1*^c^	− 1.1 ± 1^t^	− 0.3 ± 1^c^	− 0.3 ± 1^m,p,c,a,g^	− 0.5 ± 1*^c^	− 1 ± 1^t,c^	0.3 ± 1.1*^m,t,p,a,g^	− 1.5 ± 0.8 ^t,p,a^	− 0.5 ± 0.9*^c^	− 0.8 ± 0.9^m,t,c^	− 0.3 ± 1.1*^c^	− 1.4 ± 1^t,a^
BMI Z-Score	− 0.3 ± 1.1*^c^	− 1 ± 0.9 ^t,a^	− 0.3 ± 1.1^c^	− 0.4 ± 1^m,p,c,g^	− 0.4 ± 1*^c^	− 0.8 ± 0.8^t^	0.3 ± 1.1*^m,t,p,a,g^	− 1.1 ± 0.7^t,a^	− 0.4 ± 0.9^c^	− 0.6 ± 0.7^m,c,g^	− 0.4 ± 1.2*^c^	− 1.2 ± 1.1^t,a^
DB 25OHD (nmol/L)	46.7 ± 27^m^	50.2 ± 22.5	51.7 ± 19.2 m^,a^	56.6 ± 33.1^c^	45.9 ± 23^m^	47.8 ± 32.8	48.4 ± 27.3^ m^	46.2 ± 30^t^	42 ± 22.3*^,t^	50.8 ± 18.9	36.5 ± 17.4*^t,c,p,g^	56.3 ± 16.8
**Girls**												
Age (years)	10.6 ± 2.9*^t,p,c^	11.5 ± 3	12 ± 3 g	11.2 ± 3.5	12.7 ± 3.7^m,g,a^	11.8 ± 3.4	11.9 ± 2.5^ g^	12.3 ± 3.9^m,a^	10.8 ± 3^p^	11 ± 3^c^	11.4 ± 2.7*^p^	10.4 ± 2.7^c^
Height Z-Score	− 0.3 ± 1*^c^	− 0.5 ± 1^t,c^	0.1 ± 0.9 ^p,a^	0.1 ± 1^m,p,c,a,g^	− 0.5 ± 1^m,t,c^	− 0.6 ± 0.9^t,c^	0.2 ± 0.9*^p,a,g^	− 1 ± 0.9^m,p,t,g^	− 0.4 ± 0.9*^m.t,c^	− 0.8 ± 0.9^t^	0.2 ± 0.9*^p,a^	− 0.5 ± 0.8^t,c^
Weight Z-Score	− 0.5 ± 0.9*	− 0.9 ± 1^t^	− 0.3 ± 0.9^p^	− 0.2 ± 1.1^m,p,c,a,g^	− 0.7 ± 0.9^m,t,c^	− 0.9 ± 0.8^t^	− 0.2 ± 1*^p^	− 1.1 ± 0.8^t,a^	− 0.4 ± 1*	− 0.7 ± 0.9^t,c^	− 0.2 ± 0.9*^p^	− 1 ± 0.9^t^
BMI Z-Score	− 0.4 ± 1*	− 0.8 ± 1.1^t^	− 0.5 ± 1	− 0.3 ± 1.1^m,p,c,g^	− 0.6 ± 0.9*	− 0.8 ± 0.8^t^	− 0.3 ± 1*	− 0.8 ± 0.7^t^	− 0.3 ± 1	− 0.5 ± 0.9^m^	− 0.4 ± 1.1*	− 1 ± 1^t,a^
DB 25OHD (nmol/L)	41.5 ± 24.1^m^	45.9 ± 21.4	46.7 ± 17.2^m,p^	50.1 ± 25.6	38.2 ± 15.4*^t^	46.8 ± 30.9	43.3 ± 22.1^m^	45.2 ± 28.7	41 ± 17.6*^m^	47.8 ± 19.9	33.7 ± 18.7*^t,c,a,g^	49.4 ± 15.2

Significant values are in [bold].

*Level of significance (*p* < 0.05) between urban and rural regions of the same state.

Interstate significances (*p* < 0.05) are marked as state initials where m = Maharashtra, a = Assam, p = Punjab, c = Chhattisgarh, t = Tamil Nadu and g = Gujarat BMI = Body mass index (kg/m^2^).

**Figure 3:**
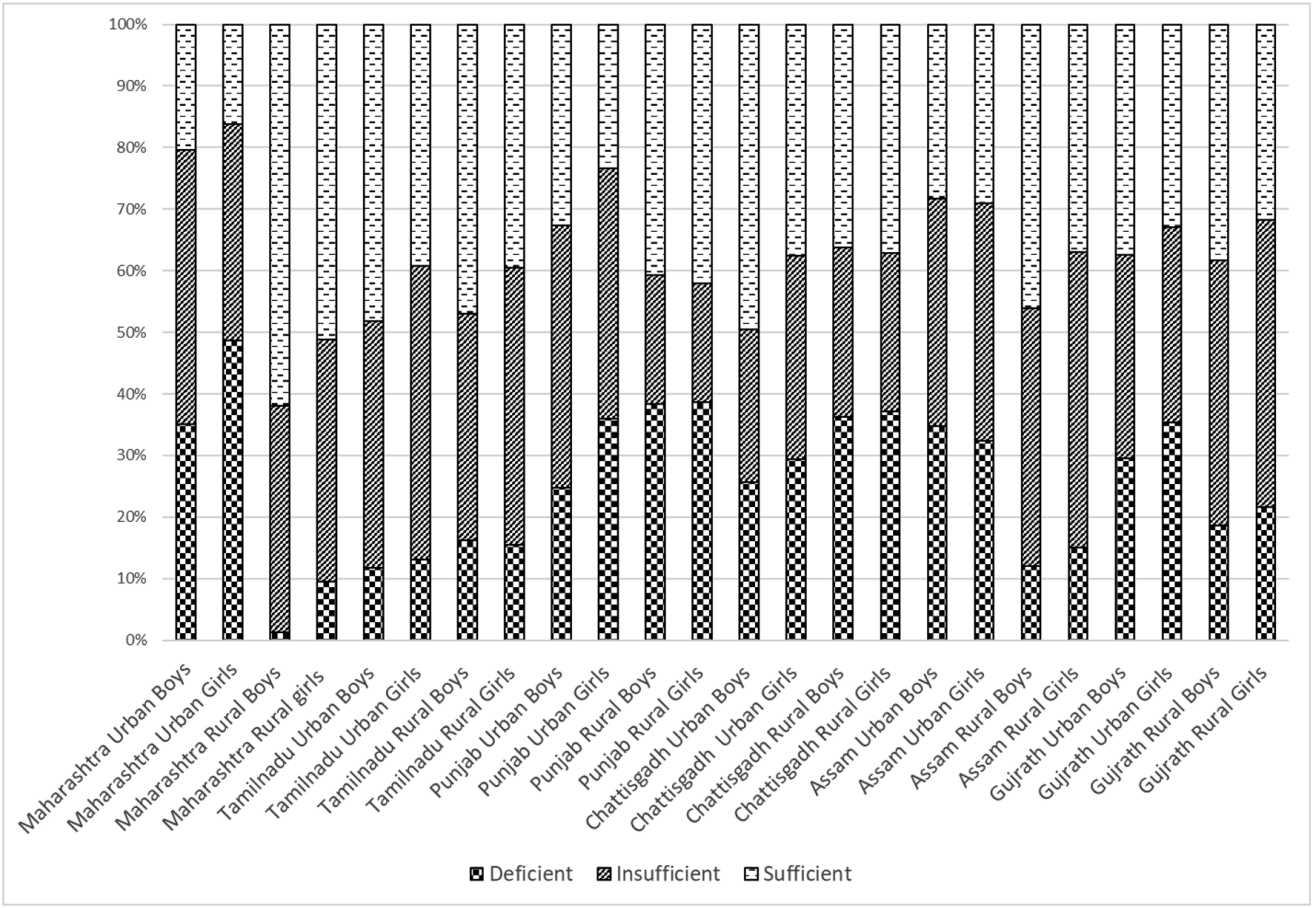
Vitamin D status (proportion deficient, insufficient, sufficient) in urban rural boys and girls from 6 Indian States.

**Figure 4:**
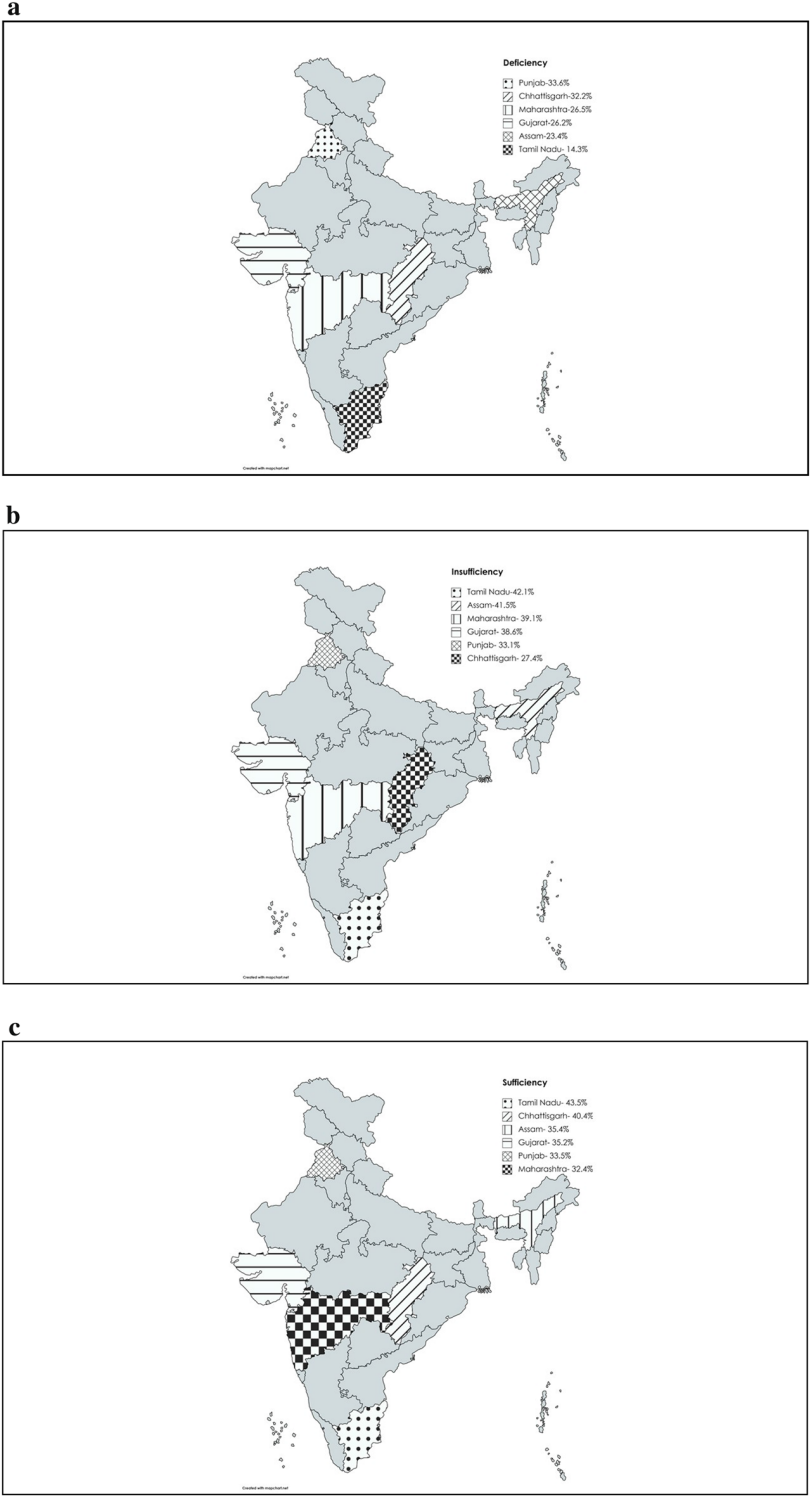
(**a**–**c**) Indian state wise prevalence of vitamin D-deficiency, insufficiency and sufficiency. (Created with mapchart.net (https://www.mapchart.net/india.html)).

Overall, only 36.8% of subjects had sufficient vitamin D concentrations (> 50 nmol/L). The prevalence of vitamin D deficiency (< 30 nmol/L) was estimated to be 26.2% while that of insufficiency (30–50 nmol/L) was 37%. If higher cut-offs as suggested by the endocrine society clinical practice guidelines^22^ only 10% of subjects had sufficient vitamin D concentrations (> 75 nmol/L), 63% were deficient (< 50 nmols/L) and 27% were insufficient (between 50 and 75 nmols/L). Gender-wise, state-wise and area-wise proportion of vitamin D deficiency/ insufficiency is illustrated in Fig. 3. Vitamin D deficiency was significantly higher in girls than boys (28.4% vs 23.9%) and higher in the urban population than rural (30.2% vs 21.6%) (*p* < 0.05 for all). However, the proportion of insufficiency by gender and rural/urban area was similar (*p* > 0.05). Figure 4a–c illustrates state-wise prevalence of deficiency, insufficiency and sufficiency of 25(OH)D_3_. Overall, the highest proportion of vitamin D sufficiency was found amongst subjects of Tamil Nadu which was significantly different from all other states included in the study except Chhattisgarh. The highest proportion of vitamin D insufficiency plus deficiency (< 50 nmol/L) was found in subjects of Maharashtra which was similar to that of other states (Punjab, Assam and Gujarat) except Tamil Nadu and Chhattisgarh.

On survey weighting, 20% population was vitamin D deficient, and 38% population was insufficient (Table 2). Table 3 represents survey-weighted descriptive characteristics of a subset of the population where along with anthropometry, body composition and dietary assessments were performed. The majority of the population belonged to the middle or lower socio-economic class as per the modified Kuppuswami’s classification^23^. The majority of children (66%) played in the sun for more than 30 min/day. Body composition parameters such as BMI Z-score, TSFT and body fat percentage Z-scores were significantly higher among the vitamin D deficient (VDD) group indicating these children were heavier than in the other two categories. Energy intakes of the VDD group were also significantly higher than the other two groups (*p* < 0.05). Calcium density per 100 kcal was also significantly higher among VDD groups as compared to insufficient and sufficient vitamin D concentration groups (*p* < 0.05).

**Table 2:** Survey Weighted parameters (age, anthropometric Z scores and Vitamin D concentrations and prevalence of deficiency/insufficiency and sufficiency) of the study population as per vit D deficiency category (n = 2500, all values survey-weighted).

Vitamin D status	Survey weighted—anthropometric and vitamin D deficiency parameters			
	Deficient (< 30 nmol/L)	Insufficient (30–50 nmol/L)	Sufficient (> 50 nmol/L)	Total
N^a^	8,822,572	16,346,782	18,037,571	43,206,925
Age (years)^b^	12.5 ± 2.2	12.4 ± 2.2	12.8 ± 2.2	12.6 ± 2.2
Height Z-Score^b^	− 0.3 ± 1.1	− 0.5 ± 1.1	− 0.5 ± 1.1	− 0.5 ± 1.1
Weight Z-Score^b^	− 0.5 ± 1.1	− 0.7 ± 1	− 0.7 ± 1	− 0.7 ± 1
BMI Z-Score^b^	− 0.6 ± 1.1	− 0.6 ± 1	− 0.7 ± 1	− 0.6 ± 1
Vitamin D (nmol/L)^b^	20.7 ± 7.2	39.7 ± 5.8	69.6 ± 19.5	48.3 ± 23.6
Percent population	20	38	42	100

^a^Survey weighted descriptive as per Indian population Census 2011^21^.

^b^All the groups were significantly different from each other, *p* < 0.05.

**Table 3:** Comparison of socio-economic status, anthropometry, body composition and lifestyle factors of children and adolescents based on vitamin D concentrations (n = 669, all values are survey-weighted^a^).

Parameters^b^	Survey weighted ^a^			
	Deficient (< 30 nmols/L)	Insufficient (30–50 nmols/L)	Sufficient (> 50 nmols/L)	Total
N^a^	3,609,460	6,137,148	5,570,475	15,317,083
Age in years	12.8 ± 1.9	13 ± 2	13.6 ± 2.2	13.2 ± 2.1
Height Z-Score	− 0.1 ± 0.9	− 0.4 ± 1	− 0.5 ± 1.1	− 0.3 ± 1
Weight Z-Score	− 0.3 ± 0.9	− 0.6 ± 0.7	− 0.7 ± 0.9	− 0.6 ± 0.8
BMI Z-Score	− 0.4 ± 0.9	− 0.6 ± 0.7	− 0.7 ± 0.7	− 0.6 ± 0.8
TSFT (mms)	12 ± 7	9 ± 6	9 ± 5	10 ± 6
TSFT Z-Score	− 0.6 ± 1.3	− 1.1 ± 1.2	− 1.3 ± 1.2	− 1 ± 1.3
Fat percent	19 ± 10	17 ± 9	16 ± 9	17 ± 9
Fat percentage Z-score	− 0.4 ± 1.0	− 0.6 ± 0.9	− 0.7 ± 0.9	− 0.6 ± 0.9
Muscle mass percent	77 ± 10	79 ± 9	80 ± 9	79 ± 9
Muscle mass percent Z-score	0.3 ± 1.0	0.6 ± 0.9	0.7 ± 0.8	0.6 ± 0.9
Energy (kcal/day)	1940 ± 620	1750 ± 600	1860 ± 630	1830 ± 620
Proteins (g/day)	49 ± 18	44 ± 18	47 ± 19	46 ± 19
Fat (g/day)	62 ± 26	53 ± 25	56 ± 27	56 ± 27
Calcium (mg/day)	630 ± 380	460 ± 320	490 ± 330	510 ± 340
Calcium-Density (mg/100 kcal)	30 ± 13	27 ± 14	26 ± 13	27 ± 13
Phosphorous(mg/day)	1100 ± 430	960 ± 380	1000 ± 410	1000 ± 400
Calcium: Phosphorus	0.5 ± 0.2	0.5 ± 0.2	0.5 ± 0.2	0.5 ± 0.2
LES (% N)	45	49	54	50
MES (% N)	48	45	40	46
Playing in sun (% N)	65	65	67	66

*TSFT* Triceps skinfold thickness, *LES* lower socioeconomic class, *MES* middle socioeconomic class.

^a^Survey weighted descriptive as per Indian population Census 2011^21^.

^b^All the groups were significantly different from each other, *p* < 0.05.

Tables 4 and 5 presents a multinomial logistic regression model describing factors affecting vitamin D deficiency and vitamin D insufficiency with reference to vitamin D sufficiency. Regression analysis was performed using two separate models. In Model 1, area of residence (urban/rural) was used as one of the predictors and in Model 2 the Indian states were used as predictors with Maharashtra considered as the reference category (due to the lowest proportion of vitamin D sufficiency) along with other lifestyle and body composition predictors (body fat % Z score and BMI Z score were collinear, hence latter was used). We observed that younger age [OR = 0.75 (0.67–0.84), *p* < 0.05], female gender [OR = 2.18 (1.37–3.47), *p* < 0.05], overweight status [OR = 2.38 (1.25–4.55), *p* < 0.05], urban residence [OR = 2.03 (1.24–3.30), *p* < 0.05], and residing in Maharashtra state were significant factors contributing to vitamin D deficiency (*p* < 0.05). Factors such as socio-economic class, sunlight exposure, dietary calcium intakes (adjusted for 100 kcal/day) and calcium to phosphorus ratio and triceps skinfold thickness did not affect deficiency or insufficiency status in the study population.

**Table 4:** Multinomial logistic regression to determine predictors of vitamin D status in subset study population, n = 669 (Model 1: demographic, anthropometric, body composition and lifestyle parameters including urban/rural residence).

Independent variables^#^	Model I			
	Vit D deficient		Vit D insufficient	
Distribution (%N)		19%		34%

	OR	95% CI	OR	95% CI
Age (years)	0.75*	0.67–0.85	0.78*	0.78–0.93
Calcium density mg/100 kcal	1.00	0.96–1.05	0.98	0.98–1.06
Calcium:phosphorus ratio	0.81	0.04–16.79	0.01	0.01–1.58
Girls	2.18*	1.37–3.47	0.89	0.89–1.84
Boys	Reference		Reference	
Low socio class	0.53	0.22–1.28	0.40	0.40–1.92
Medium socio class	0.67	0.27–1.64	0.60	0.60–2.98
High socio class	Reference		Reference	
No playing in Sun	1.0	0.63–1.63	0.88	0.88–1.88
Playing in Sun	Reference		Reference	
Overweight BMI Z	2.39*	1.25–4.55	0.66	0.66–2.24
Normal weight BMI Z	Reference		Reference	
High TSFT Z	2.37	0.81–6.89	0.51	0.51–4.62
Low TSFT Z	Reference		Reference	
Urban	2.03*	1.25–3.30	0.82	0.82–1.75
Rural	Reference		Reference	
Constant	2.33		2.19	
Nagelkerke R^2^	0.13		0.13	
Correct predicted %				52

Significant values are in [bold].

*TSFT* triceps skin fold thickness, *Z* Z-scores.

Level of Significance—*p < 0.05.

^#^Variables are presented as OR (95% CI).

**Table 5:** Multinomial logistic regression to determine predictors of vitamin D status in subset study population, n = 669 (Model 2: demographic, anthropometric, body composition and lifestyle parameters and state of residence).

Independent variables^#^	Model 2			
	Vit D deficient		Vit D insufficient	
Distribution (%N)		19%		34%

	OR	95% CI	OR	95% CI
Age (years)	0.87*	0.76–1.00	0.91*	0.83–0.99
Calcium density mg/100 kcal	1.00	0.95–1.05	1.01	0.97–1.05
Calcium:phosphorus ratio	1.45	0.05–46.91	0.31	0.02–5.18
Girls	2.27*	1.36–3.77	1.29	0.88–1.87
Boys	Reference		Reference	
Low socio class	0.70	0.26–1.88	0.96	0.42–2.21
Medium socio class	0.65	0.23–1.79	1.35	0.58–3.14
High socio class	Reference		Reference	
No playing in Sun	0.63	0.37–1.09	1.14	0.76–1.72
Playing in Sun	Reference		Reference	
Overweight BMI Z	2.44*	1.18–5.05	1.22	0.65–2.30
Normal weight BMI Z	Reference		Reference	
High TSFT Z	1.12	0.33–3.81	0.95	0.29–3.07
Low TSFT Z	Reference		Reference	
Gujarat	0.54	0.24–1.26	0.82	0.38–1.8
Tamil Nadu	0.07*	0.03–0.16	0.35*	0.19–0.67
Chandigarh	0.04*	0.01–0.10	0.13*	0.06–0.26
Raipur	0.03*	0.01–0.09	0.30*	0.16–0.56
Assam	0.24*	0.10–0.57	0.55	0.26–1.16
Maharashtra	Reference		Reference	
Constant		2.22		2.07
Nagelkerke R^2^	0.30		0.30	
Correct predicted %				59

Significant values are in [bold].

*TSFT* triceps skin fold thickness, *Z* Z-scores.

Level of Significance—*p < 0.05.

^#^Variables are presented as OR (95% CI).

## Discussion

To the best of our knowledge, this is the first multicentre study in which the vitamin D status of Indian children and adolescents was assessed using the LCMS/MS method. In this multistate study on Indian children and adolescents of age 5–18 years, only around 42% were vitamin D sufficient (25(OH)D > 50 nmol/L). Thus, the combined prevalence of vitamin D deficiency and insufficiency was 58% [(25OH) D < 50 nmol/L, 20% deficient and 38% insufficient]. The mean Vitamin D concentration of 25(OH)D_3_ was below 50 nmol/L. If cut-offs as suggested by the endocrine society guidelines^22^ were used, only 10% of subjects had sufficient vitamin D concentrations (> 75 nmol/L). We found interstate differences in the prevalence of vitamin D deficiency. The western state of Maharashtra had the highest prevalence of deficient and insufficient children and adolescents, while those from the southern state of Tamil Nadu and the central state of Chhattisgarh were the most vitamin D sufficient. Further, the prevalence of vitamin D deficiency was significantly higher in girls than boys (28.4% vs 23.9%) and in the urban population than in the rural (30.2% vs 21.6%). We also report that younger age, female gender, overweight and urban residence were significant factors contributing to vitamin D deficiency while factors like socioeconomic status, sunlight exposure, dietary calcium intakes, dietary calcium to phosphorus ratio and triceps skinfold thickness did not have a significant impact on vitamin D status. Despite Vitamin D deficiency/insufficiency, none of the children on clinical examination had musculoskeletal symptoms (Data not shown).

High prevalence of vitamin D deficiency has been noted worldwide. Even developed countries like the United States of America (USA), Canada and Europe report that 5.9%, 7.4% and 13% of their population is deficient and 24%, 37% and 40%, respectively, are insufficient^24^. Another American study on adolescents of age 11–18 years reports concentrations < 50 nmol/L in 42% of subjects while a study in the age group of 1–21 years reported deficiency plus insufficiency in 70% of subjects^25^. Studies from Germany (deficiency in 12.5% and insufficiency in 32.7–33.5%) and England (severe deficiency < 25 nmol/L in 12–16% in 4–10 years and 20–24% in 11–18 years) have also reported vitamin D deficiency in the paediatric age group^26^. Studies from sun-rich countries like Ethiopia and Saudi Arabia have also reported 42% and 99.8% prevalence of vitamin D deficiency respectively, in the paediatric population^27,28^. Low and middle-income countries like Pakistan, Afghanistan and Tunisia have also reported 25(OH)D_3_ concentrations of < 30 nmols/L in > 20% of the population^24^.

Various studies in different parts of India have reported widespread prevalence of vitamin D deficiency in all age groups. It is estimated that the prevalence of deficiency is 62–95.7% in new-borns and breast-feeding groups (0–6 months), 46–80% in 6–60 months of age and 37.8–97.5% in 5–20-year-old children^13^. A systemic analysis reports the prevalence of vitamin D deficiency in children of age 5–20 years between 37.8 and 97.5%^13^. Similar to our results, another systematic review on adolescent girls in India reports the pooled prevalence of vitamin D deficiency to be 25.7%^29^, while a narrative review suggests that the prevalence of vitamin D deficiency in Indian children varies from 50 to 90%^30^. In our studies from Western India, we have found that the prevalence of vitamin D deficiency in children and adolescents ranged from 30 to 98%^31,32^.

In the present study, participants living in Maharashtra (Western India) had the highest prevalence of vitamin D deficiency compared to participants living in other states and state of residence was a significant predictor of vitamin D deficiency. However, no relationship between latitude and vitamin D deficiency has been reported by various international studies in children and adults^33–35^. We also report that Tamil Nadu and Chhattisgarh had significantly different vitamin D sufficiency proportions than Maharashtra (18.5° N), and they geographically lie south (13° N) and north (21.2° N) of Maharashtra respectively. In various multicentre studies performed in geographically vast countries with variable weather conditions like China and Australia, no relationship of vitamin D deficiency with latitude has been found, just as in our study^36,37^. Prevailing weather conditions including air pollution levels, differences in dietary habits and season are regarded as potential confounders that complicate the relationship between latitude and prevalence of vitamin D deficiency.

The comparison of prevalence estimates by various Indian studies in the states where our study was conducted is illustrated in Table 6^38–43^. These variations in results may be due to sampling bias (some of these studies were conducted in the hospital setting), age group of samples studied, sample size and composition, socio-economic status of study populations, cut-offs for vitamin D deficiency and insufficiency and methods of vitamin D estimation. Data from these studies are also limited by the use of immunoassay methods for vitamin D assessment. We also attempted to compare our results with the data from the comprehensive national nutritional survey; (the report presents weighted data)^44^, the trends in Maharashtra, Gujarat and Punjab in the CNNS and current study were similar. The aforementioned variations in the estimates of vitamin D deficiency underline the importance of a study such as the present one where similar methods of sampling participants, assessment of vitamin D concentrations and similar cut-offs for deficiency and insufficiency have been used.

**Table 6:** Comparison of vitamin D status between present study and earlier Indian studies:

State	N	Age group	Setting	Method of 25(OH)D assay	Vitamin D deficiency	Vitamin D insufficiency	Our study	
							Deficiency	Insufficiency
Tamil Nadu^38^	230	6 months–18 years	Hospital	Immunochemiluminometric assay	37.4%	24.8%	14.3%	42.1%
Gujarat^39^	41	0–20 years	Community	Electrochemiluminescence immunoassay	61.4%		64.8%	
Punjab^40^	338	3 months–12 years	Hospital	Electrochemiluminescence immunoassay	40.2%	25.4%	33.6%	33.1%
Maharashtra^41^	359	6–12 years	Community	ELISA	24%	71%	28.5%	39.1%
Assam^42^	500	8–14 years	Community	Radioimmunoassay	8.4%	14.2%	23.4%	41.5%
Chhattisgarh^43^	101	2–18 years	Thalassaemic children	Electrochemiluminescence	2%	50.5%	32.2%	27.4%

On multinomial logistic regression analysis, we report a significantly lower risk of vitamin D deficiency with increasing age. The Healthy Lifestyle in Europe by Nutrition in Adolescence (HELENA) study also reports a steady increase in 25(OH)D_3_ concentrations with increasing age which were significant in girls^45^. Another study also found higher 25(OH)D_3_ serum concentrations in girls’ post-menarche as compared to pre-menarche due to an increase in the vitamin D binding protein because of higher oestrogen concentrations^46^. We however report that vitamin D deficiency was more prevalent in girls than in boys, similar to that documented by Marwaha et al from Northern India^47^. Several factors may be responsible including the type of clothing, lesser participation in outdoor activities leading to decreased cutaneous vitamin D synthesis, etc. Also, in rural India, social factors like boys being allowed greater freedom to play outdoors and preference in terms of diet could impact vitamin D status in girls^48^.

We also report that overweight subjects and urban populations are at a higher risk of development of vitamin D deficiency. The inverse relationship of BMI and vitamin D concentrations is well known and is explained by decreased bioavailability of vitamin D (cutaneous synthesis as well as dietary) due to its sequestration into a larger pool of adipose tissue^49^. The higher prevalence in urban children may be explained by a higher prevalence of obesity and a sedentary lifestyle. An Indian study has also reported a lower prevalence of 25(OH)D deficiency in rural subjects compared to that of urban subjects attributing it to occupation, dress code and duration of exposure to sunlight^50^. Interestingly, in a Chinese study, rural girls despite having higher exposure to UV rays than urban girls, had significantly lower 25(OH)D_3_. The investigators attributed this finding to the lower calcium intake in rural girls^51^. We noted that socioeconomic status and dietary factors like calcium intake and calcium to phosphorus ratio were not significant in determining vitamin D deficiency. A study from Delhi concluded that although girls of lower socioeconomic strata had higher daily sun exposure, a higher percentage of body surface area exposed and low dietary calcium intake, the prevalence of vitamin D deficiency was significantly higher among girls belonging to upper socio-economic status due to their significantly higher BMI and body fat percentage^52^.

Mandlik et al. in their study on Maharashtrian school children of age 6–12 years identified the duration of sunlight exposure as a significant determinant of vitamin D concentrations^41^. Wakayo et al. in a study from Ethiopia also report that duration of sun exposure was a significant determinant of Vitamin D status in 11–18-year-olds^27^. However, in our study, sunlight exposure was not found to be a significant predictor of vitamin D status. This is probably because urban children and adolescents are more likely to remain indoors in comparison to rural children who are reported to have higher sunlight exposure^53^. Thus, as the location of residence (urban/rural) likely represented the duration of sunlight exposure, it was possibly not an independent predictor of vitamin D status.

The strengths of our study are that ours was a multistate study, the same team was involved in data collection at all sites and analysis of 25(OH)D_3_ concentrations was performed using a standardized protocol in one laboratory on the same equipment. To the best of our knowledge, ours is the only epidemiological Indian study where 25(OH)D_3_ concentrations have been estimated using the LCMS/MS. Our study is limited by the fact that it was a cross-sectional study (associations and no causation could be studied), we did not collect data related to environmental conditions, UVB radiation and pollution. Although the study was from different geographical locations, most Indians have a similar Fitzpatrick skin type (V or VI), hence we did not control for the variation in skin colour^54–56^. Also, this being a school-based study and 25(OH)D_3_ estimates were performed from dried blood spots, we were not able to assess other blood parameters such as serum calcium, phosphorus, parathyroid hormone, etc. Moreover, as previous studies suggest that for most Indians located between 8.4° and 37.6° N, the time required for recommended vitamin D synthesis is similar, including in the winter months, we did not control for the seasonal variation. We used a validated protocol for assessing 25(OH)D_3_, however, our study was not a part of a program to standardize laboratory measurements of vitamin D status. We studied children in the months of June to March (the typical school year in India is from June to April) which are the summer (rains) and winter seasons. Thus, children were not measured in the same season. However, previous studies suggest that for most Indians located between 8.4° and 37.6° N, the time required for recommended vitamin D synthesis for skin type is similar, including in the winter months (10–45 min at noon)^57^. Lastly, we have not presented data related to functional outcomes of vitamin D deficiency and additional data could be collected only on a subset. Thus, there is an urgent need to develop programs to educate the population about the prevailing status of vitamin D deficiency and for vitamin D supplementation or food fortification, particularly in children and adolescents.

To conclude, we report vitamin D status in Indian children and adolescents using standardized methodology and the LCMS/MS method. More than half the children and adolescent population in our multistate study (which included urban and rural subjects) were vitamin D deficient or insufficient. We describe interstate and urban/ rural differences in vitamin D status. Our study reinforces that vitamin D deficiency is a significant public health concern in Indian children and adolescents, especially in girls, in the young, the urban and the obese.

## Data Availability

Some datasets generated during and/or analyzed during the current study are not publicly available but are available from the corresponding author on reasonable request.
